# Combined anticancer therapy with imidazoacridinone analogue C‐1305 and paclitaxel in human lung and colon cancer xenografts—Modulation of tumour angiogenesis

**DOI:** 10.1111/jcmm.17430

**Published:** 2022-06-14

**Authors:** Marta Świtalska, Beata Filip‐Psurska, Magdalena Milczarek, Mateusz Psurski, Adrianna Moszyńska, Aleksandra M. Dąbrowska, Małgorzata Gawrońska, Karol Krzymiński, Maciej Bagiński, Rafał Bartoszewski, Joanna Wietrzyk

**Affiliations:** ^1^ Department of Experimental Oncology Hirszfeld Institute of Immunology and Experimental Therapy Wrocław Poland; ^2^ Department of Biology and Pharmaceutical Botany Medical University of Gdansk Gdańsk Poland; ^3^ Department of Immunology, Faculty of Biochemistry, Biophysics and Biotechnology Jagiellonian University Kraków Poland; ^4^ Faculty of Chemistry University of Gdańsk Gdańsk Poland; ^5^ Department of Pharmaceutical Technology and Biochemistry, Faculty of Chemistry Gdansk University of Technology Gdańsk Poland

**Keywords:** 5‐dimethylaminopropylamino‐8‐hydroxytriazoloacridinone, angiogenesis, C‐1305, colon cancer, combined treatment, imidazoacridinone, lung cancer, paclitaxel

## Abstract

The acridanone derivative 5‐dimethylaminopropylamino‐8‐hydroxytriazoloacridinone (C‐1305) has been described as a potent inhibitor of cancer cell growth. Its mechanism of action in in vitro conditions was attributed, among others, to its ability to bind and stabilize the microtubule network and subsequently exhibit its tumour‐suppressive effects in synergy with paclitaxel (PTX). Therefore, the objective of the present study was to analyse the effects of the combined treatment of C‐1305 and PTX in vivo. In addition, considering the results of previous genomic analyses, particular attention was given to the effects of this treatment on tumour angiogenesis. Treatment with C‐1305 revealed antitumor effect in A549 lung cancer cells, and combined treatment with PTX showed tendency to anticancer activity in HCT116 colon cancer xenografts. It also improved tumour blood perfusion in both tumour models. The plasma level of CCL2 was increased and that of PDGF was decreased after combined treatment with C‐1305 and PTX. The experimental results showed that the levels of FGF1, TGF‐β and Ang‐4 decreased, whereas the levels of ERK1/2 and Akt phosphorylation increased in HCT116 tumour tissue following combined treatment with both drugs. The results of in vitro capillary‐like structure formation assay demonstrated the inhibiting effect of C‐1305 on this process. Although previous in vitro and in vivo studies suggested a positive effect of C‐1305 on cancer cells, combined treatment of HCT116 human colon and A549 lung cancer cells with both PTX and C‐1305 in vivo showed that the antitumor activity was restricted and associated with the modulation of tumour angiogenesis.

## 1 INTRODUCTION

**FIGURE 1:**
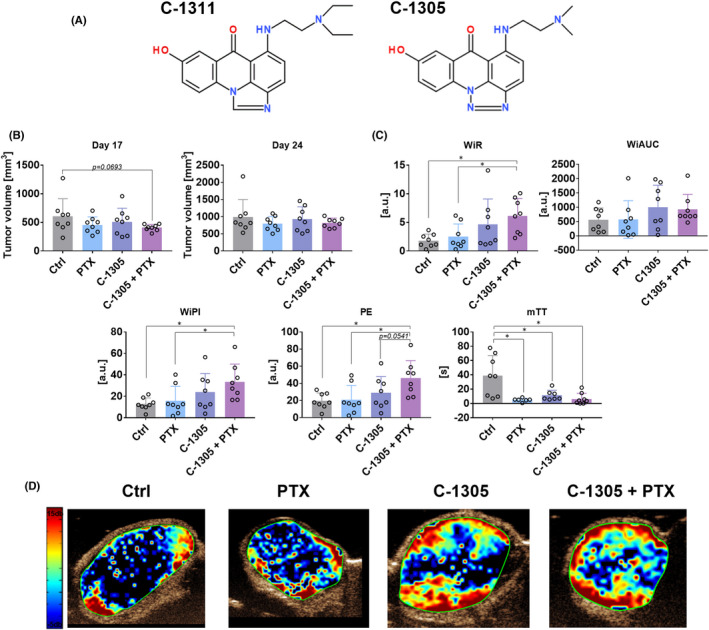
Tumour burden and blood flow in mice bearing human HCT116 colon cancer cells and treated with C‐1305 and PTX. (A) The chemical structures of C‐1305 and C‐1311. (B) Tumour volume measured on Days 17 and 24. Tumour growth kinetics is presented in Figure S1. (C) Parameters describing blood flow in tumour tissue. WiR, wash‐in‐rate; WiAUC, wash‐in area under the curve; WiPI, wash‐in perfusion index; PE, peak enhancement; mTT, mean transit time. (D) Representative images of WiR. HCT116 cells were injected subcutaneously. Treatment was started on the 10th day with intraperitoneal injections of 10 mg/kg PTX and 30 mg/kg C‐1305. Then, PTX was injected once every week and C‐1305 was injected 5 days a week for a period of 3 weeks. For blood flow analysis on the 18th day, anaesthetised mice were injected with contrast agent and ultrasound detection was performed. *N* = 8; statistical analysis: Sidak's multiple comparisons test. *p < 0.05

The inability to decipher molecular mechanisms underlying the molecule's biological activity is often the limiting factor for its further therapeutic application. Hence, although a large number of anticancer molecules entered the drug pipelines, only a few of them reached clinical trials. In contrast, many promising compounds were left behind due to an inability to explain complex mechanisms determining their biological activities. Although, the increasing affordability of genome‐wide analysis technologies enables in vitro revalidation of molecular pathways associated with the activity of these neglected drug candidates, functional verification of these activities in vivo is also required. Such an omitted molecule, 5‐dimethylaminopropylamino‐8‐hydroxytriazoloacridinone (C‐1305),^1^ was synthesized for the first time during the development of synthetic ligands that can effectively interact with DNA^2^ and expressed a strong cytotoxic and cytostatic effect both in vitro^3^ and in vivo (solid tumour models) conditions.^4^ However, despite extensive research, there have been contradictory reports in the literature regarding the mechanism of apoptosis induced by C‐1305. Although the ability of C‐1305 to covalently bind DNA, preferably in guanine‐rich regions was reported,^5^, ^6^, ^7^ no association was found between the ability of C‐1305 to crosslink DNA and its biological activity.^8^ Further studies focused on the evaluation of the effect of C‐1305 on the topoisomerase II activity showed that treatment with C‐1305 led to the formation of low levels of potent cleavable complexes that are selectively toxic toward tumour cells with impaired p53 function.^9^ In addition, cancer cells with such characteristics were inhibited in the G_2_/M cell cycle phase, which was followed by elevated phosphorylation of CDK1 at the inhibitory sites (Thr14/Tyr15) and increased phosphorylation of pRb protein.^10^ Interestingly, breast cancer cells that overexpress BRCA1 protein showed resistance to C‐1305, which was overcome by the inhibition of PARP‐1 expression.^11^ Moreover, mouse cells lacking PARP‐1 were found to be extremely sensitive to C‐1305.^6^ However, such a phenomenon is not observed for DNA topoisomerase II inhibitors and can be related to the reactivation of p53 pathway.^12^ Some studies demonstrated that C‐1305 can induce apoptosis in cells with internal tandem duplication mutation of FMS‐like receptor tyrosine kinase (FLT3), suggesting that FLT3 can be a new target molecule for C‐1305.^13^ C‐1305 has also been identified as a potential inhibitor of inositol‐requiring enzyme 1‐α, one of the sensors in the unfolding protein response that alleviates endoplasmic reticulum stress in cells and functions to promote cell survival.^14^ We have recently used an in vitro genome‐wide approach to determine transcriptomic signatures of C‐1305 cytotoxicity in human in lung and colon cancer cell lines and identified this molecule as the first microtubule‐stabilizing compound that also functions as a topoisomerase II inhibitor.^1^ Notably, the binding site of C‐1305 with microtubules, as well as the kinetics of this binding process, was different from that of paclitaxel (PTX), and thus, these compounds do not compete with each other in their mechanism of action.^1^ Importantly, the potential effectiveness of isosteric analogue of C‐1305, 5‐diethylaminoethylamino‐8‐hydroxyimidazoacridinone (C‐1311; Symadex™, NSC‐645809),^2^ in combination with PTX for the treatment of bladder cancer has been demonstrated as well.^2^, ^15^, ^16^, ^17^, ^18^, ^19^ The structural formulas of C‐1305 and C‐1311 are shown in Figure 1A.

These reports suggested that C‐1305 combined treatment with PTX could be used to develop an effective anticancer therapeutic approach and the present study aimed to investigate the effects of combined treatment of C‐1305 and PTX in vivo. Furthermore, based on the results of previous genomic analyses, particular attention was given to determining the effects of the treatment on tumour angiogenesis, which revealed the modulating effect of C‐1305 on this process. However, despite the previous encouraging results in human colon and lung cancers when combined treatment with PTX was given, the in vivo experiments of the present study showed that the antitumor activity was restricted and associated with the modulation of tumour angiogenesis.

## 2 MATERIALS AND METHODS

Compound C‐1305 has been recently resynthesized, applying thoroughly optimized method of preparation and its purity has been precisely determined prior to investigations described here. Details concerning aspects of synthesis of C‐1305 and its spectroscopic analyses are presented in our former work.^1^

### 2.1 Cell lines

Human colon carcinoma HCT116 cell line was obtained from the American Type Culture Collection, and human lung adenocarcinoma A549 cell line from the European Collection of Authenticated Cell Cultures. All the cell lines are maintained at the Hirszfeld Institute of Immunology and Experimental Therapy, PAS, Wroclaw, Poland.

A549 cells were cultured in RPMI 1640 + OptiMEM (1:1) medium (Gibco, UK) with 5% fetal bovine serum (FBS) (HyClone Laboratories, Logan, Utah, USA) and supplemented with 2 mM L‐glutamine (Sigma‐Aldrich, Germany). HCT116 cells were cultured in McCoy's medium with L‐glutamine (Gibco, UK) supplemented with 10% FBS (HyClone Laboratories). All the culture media were supplemented with 100 units/ml penicillin (Polfa Tarchomin S.A., Poland) and 100 μg/ml streptomycin (Sigma‐Aldrich). All the cell lines were grown at 37°C in the atmosphere humidified with 5% CO_2_.

Primary human umbilical vein endothelial cells (#ZHC‐2301) pooled from 10 independent donors were obtained from Cellworks (Division of Catalog Medsystems Ltd, UK) and cultured in EGM‐2 (endothelial cell growth medium‐2) BulletKit medium (Lonza, Switzerland). All experiments were conducted at passage 4 when a confluence of 80% was reached. Cells were cultured in a humidified incubator (Thermo Fisher Scientific) in T75 culture flasks (Falcon, Germany) at 37°C in an atmosphere of 5% CO_2_ before they were plating on 96‐well plates for angiogenic assays.

### 2.2 Mice

Experiments were carried out on 6‐ to 8‐week‐old female BALB/c nude mice (CByJ.Cg‐Foxn1nu/J). Each test group consisted of 8 mice. Approval for the study was obtained from the Local Ethical Committee for Animal Experiments in Wroclaw (permission number: 13/2020). All the experiments were performed according to Directive 2010/63/EU of the European Parliament and Council on the protection of laboratory animals used for scientific purposes. Mice were purchased from Charles River Laboratories. Animals were housed under specific pathogen‐free conditions and exposed to 12‐h day/night cycles with access to feed and water ad libitum at the animal facility of Hirszfeld Institute of Immunology and Experimental Therapy in Wroclaw, Poland.

### 2.3 Animal experiments scheme

Mice were injected subcutaneously on the right side of the body with A549 or HCT116 cells grown in a suspension containing 5 × 10^6^/0.15 ml phosphate‐buffered saline and 0.05 ml Matrigel (v:v, 3:1, Matrigel Basement Membrane Matrix, High Concentration). When tumours reached a volume of ~50 mm^3^ (for A549 cells) or ~160 mm^3^ (for HCT116 cells), the treatment was initiated. C‐1305 was synthesized by the Faculty of Chemistry, University of Gdansk, Poland, and verified for purity and identity using RP‐HPLC, elemental analysis, ^1^H NMR spectroscopy and HR‐MS spectrometry as described in previous studies.^1^, ^2^ After characterization studies, C‐1305 was dissolved in 5% glucose, and a single dose of 30 mg/kg of body weight was administered intraperitoneally five times a week for a period of 3 weeks. PTX (Paclitaxelum 6 mg/ml; Accord) was administered intraperitoneally as a single dose at 10 mg/kg of body weight once a week for a period of 3 weeks. The animals were observed carefully for 34 (A549) or 26 (HCT116) days and then were euthanized. Body weight and tumour growth were monitored three times a week. Tumour growth was monitored by measuring the largest diameter (a) and perpendicular diameter (b), and the tumour volume (TV) was calculated according to the formula TV (mm^3^) = 0.5 × *a* (mm) × *b* ^2^ (mm). Blood and tumour tissue were harvested during autopsy for further analyses.

### 2.4 Analysis of tumour blood perfusion

On the 18th day (HCT116 model) or 32nd day (A549 model), tumour perfusion analysis was performed using the Vevo 2100 ultrasound imaging system (VisualSonics, Ontario, Canada) and MicroMarkerTM contrast agent (VisualSonics). The contrast agent was dissolved in 1 ml of sterile 0.9% saline solution (PChO HIIET). Mice were subjected to general anaesthesia by a continuous administration of 2%–3% isoflurane (Baxter) in synthetic air (600 ml/min) and immobilized on the treatment table. The tumour was covered with air bubble‐free gel, and its central cross‐section was visualized in a transverse plane using MS250 scanhead (VisualSonics). About 100 μl of the contrast agent was administered intravenously, and the first imaging sequence (bolus) was recorded (ca. 15 fps) after the contrast signal in the tumour tissue reached a steady state (ca. 50 s). Then, contrast microbubbles within the field of view were destroyed using burst mode, and a second imaging sequence (replenishment) was recorded. After the imaging process was completed, mice were kept in a warm environment until fully awakened. Data analysis was performed using the Vevo LAB 1.7.1 Software with VevoCQ modality (VisualSonics).

### 2.5 Tissue lysate preparation

HCT116 tumours harvested from mice were frozen in liquid nitrogen and then stored at −80°C. About 300 mg of sample was collected from frozen tissues and subsequently transferred to cork‐sealed homogenizing tubes containing a homogenizing ball (Mp Biomedicals LLC.) and 500 μl of RIPA buffer containing phosphatase and protease inhibitor cocktail (1 ml of RIPA buffer + 10 μl cocktail of protease inhibitors + 10 μl cocktail of phosphatase 1 inhibitors + 10 μl of phosphatase 2 inhibitors). All reagents were purchased from Sigma‐Aldrich Chemie GmbH. Homogenization of tissues was done using the Fast Prep®‐24 MP Bio homogenizer (Mp Biomedicals) with the following settings: CP 24 × 2, 6 m/s, *t* = 40 s, and the homogenization cycle was repeated twice. The resulting suspension was incubated on ice for 20 min, then frozen in liquid nitrogen and centrifuged after thawing (4°C, 15 min, 14,000 *g*). The supernatant was transferred to clean 1.5 ml tubes (Sarstedt), centrifuged again, and the supernatant was collected into Eppendorf tubes and stored at −80°C for further analyses. Total protein concentration was determined by the modified Lowry assay (Bio‐Rad) according to the manufacturer's protocol.

### 2.6 Automated capillary western blot analysis and total protein detection

The HCT116 tumour lysates were analysed for protein phosphorylation and expression using Jess Simple Western System, an automated capillary‐based size‐sorting system (ProteinSimple). The evaluation of targeted proteins, Ang‐4 (MAB964; R&D Systems**)**, COX‐2 (AF4198; R&D Systems), p53 (MAB1355; R&D Systems), ERK1/ERK2 (MAB1576; R&D Systems), phospho‐ERK1(T202/Y204)/ERK2 (T185/Y187) (MAB1018; R&D Systems), p38α (AF8691; R&D Systems), phospho‐p38α (T180/Y182) (MAB8691; R&D Systems), Akt (2920S; Cell Signalling Technology [CST]), phospho‐Akt (Ser473) (4058S; CST), VEGFR2 (2479S; CST) and eNOS (32027S; CST), was performed according to the Jess user guide provided by ProteinSimple. The protein lysate was mixed with 0.1X sample buffer and 5X master mix reagent to achieve a total protein concentration of 2 μg/μl (COX‐2, p53, ERK1/2, phospho‐ERK1/2, p38α, phospho‐p38α) or 0.5 μg/μl (eNOS, Ang‐4, VEGFR2, Akt, phospho‐Akt) in the final sample. The mixture was denatured by heating for 5 min at 95°C. Biotinylated ladder (5 μl, protein range: 12–230 or 66–440 kDa) and each protein sample (5 μl) were loaded into individual wells of the sample plate. Antibodies were diluted with antibody diluent buffer. After the detection of targeted protein by chemiluminescence or near‐infrared (NIR) fluorescence techniques, the primary and secondary antibodies were removed from capillaries to determine the total protein content in a single run. For the quantification of total proteins, the capillaries were incubated with biotin‐labelling reagent and streptavidin‐horseradish peroxidase for the detection of biotin by chemiluminescence. Compass software (version 6.0.0; ProteinSimple) was used to program in the Jess language and analyse the experimental results. Quantification by densitometry was performed using the area of targeted proteins and normalized to the total protein. Results are expressed as fold change in the expression of proteins when compared to their expression in control.

### 2.7 Detection of proteins in mice plasma using ELISA

The plasma samples of mice injected with HCT116 and A549 tumour cells were analysed for the expression levels of VEGF, PDGF(AA), CCL2 and IL‐6 proteins by using enzyme‐linked immunosorbent assay (ELISA; Invitrogen) according to the manufacturer's protocol. For this purpose, the blood samples were collected in EDTAK2 tubes and centrifuged at 2400× *g* for 14 min at 4°C. Absorbance was recorded at 450 nm using a BioTek's Synergy H4 hybrid reader (Biokom).

### 2.8 Detection of proteins in tumour lysates using ELISA

The lysates obtained from HCT116 cell line were analysed for the expression levels of VEGF, PDGF, CCL2, FGF1, FGF2 and IL‐6 proteins by using ELISA (EIAab Science) according to the manufacturer's protocol. Absorbance was recorded at 450 nm using a BioTek's Synergy H4 hybrid reader (Biokom). Results were normalized to total protein concentration in each lysate.

### 2.9 Immunohistochemical staining

HCT116 and A549 tumours were harvested from mice on Day 26 or 34 of the experiment, respectively, and analysed using standard methods at Sorbolab Research Laboratory LLC, Poznań, Poland. Standard staining technique with haematoxylin and eosin and TUNEL assay were performed. Moreover, tumour slides were stained with anti‐Ki67, anti‐CD31, anti‐telomerase reverse transcriptase (TERT), anti‐α‐tubulin and anti‐β‐3‐tubulin antibodies (all from Thermo Fisher Scientific), counterstained with haematoxylin, and finally observed under Leica DM 200 microscope (Olympus). The results were analysed by calculating the immunoreactive score taking into account the percentage of stained cells and the intensity of the reaction product.

### 2.10 Toxicity profile of the anticancer treatment

The body weight of the experimental animals was measured thrice each week throughout the course of all the studies. Biochemical analyses were performed in Cobas C 111 analyser (Roche Diagnostics) for the molecules alanine aminotransferase, aspartate aminotransferase (ASTL), urea (UREL), creatinine (CRE2) and albumin (ALB) using reagents and procedures provided by the manufacturer.

### 2.11 Angiogenic in vitro assay

Capillary‐like structure formations were accessed using the In Vitro Angiogenesis Assay Kit (ab204726ECM625; Abcam) according to the manufacturer's protocol. Briefly, cells were passaged on matrigel‐coated 96‐well plates and treated with C‐1305 to obtain the final concentrations of 1, 5 and 10 μM, respectively. The number of capillary‐like structures was counted in each well (from six randomly selected focal fields at 20× magnification) after 8 h of incubation. The capillary structure formation was measured semiquantitatively using the Wim Tube webserver (Wimasis, 2016. WimTube: Tube Formation Assay Image Analysis Solution. Release 4.0. Available from: https://www.wimasis.com/en/products/13/WimTube). A solution containing 2 pM final concentration of vinblastine (V1377; Sigma‐Aldrich) served as a negative control.

### 2.12 Statistical analysis

Statistical analysis was performed using GraphPad Prism 7.1 software. The distribution analysis of data was carried out using the Shapiro–Wilk data normality test. Specific tests were used for data analysis, and the details are presented in the figure legends. Differences between the groups were statistically significant at *p* < 0.05.

## 3 RESULTS

### 3.1 Anticancer activity of C‐1305 combined with PTX in human colon HCT116 and lung A549 cancer models

To justly evaluate the anticancer activity of C‐1305 combined with PTX in vivo, the very same lung cancer and colon cell lines (A549 and HTC 116, respectively) were used as in previous genome‐wide transcriptomic in vitro approach.^1^ Although these cancer cell lines were initially chosen based on previously reported sensitivity to C‐1305,^4^, ^9^, ^20^ their selection for the current study was determined by the ability to impartially compare results between in vitro and in vivo data. Furthermore, C‐1311 significant activity against murine and human colorectal cancer cells was reported in vitro and in vivo as well.^16^

**FIGURE 2:**
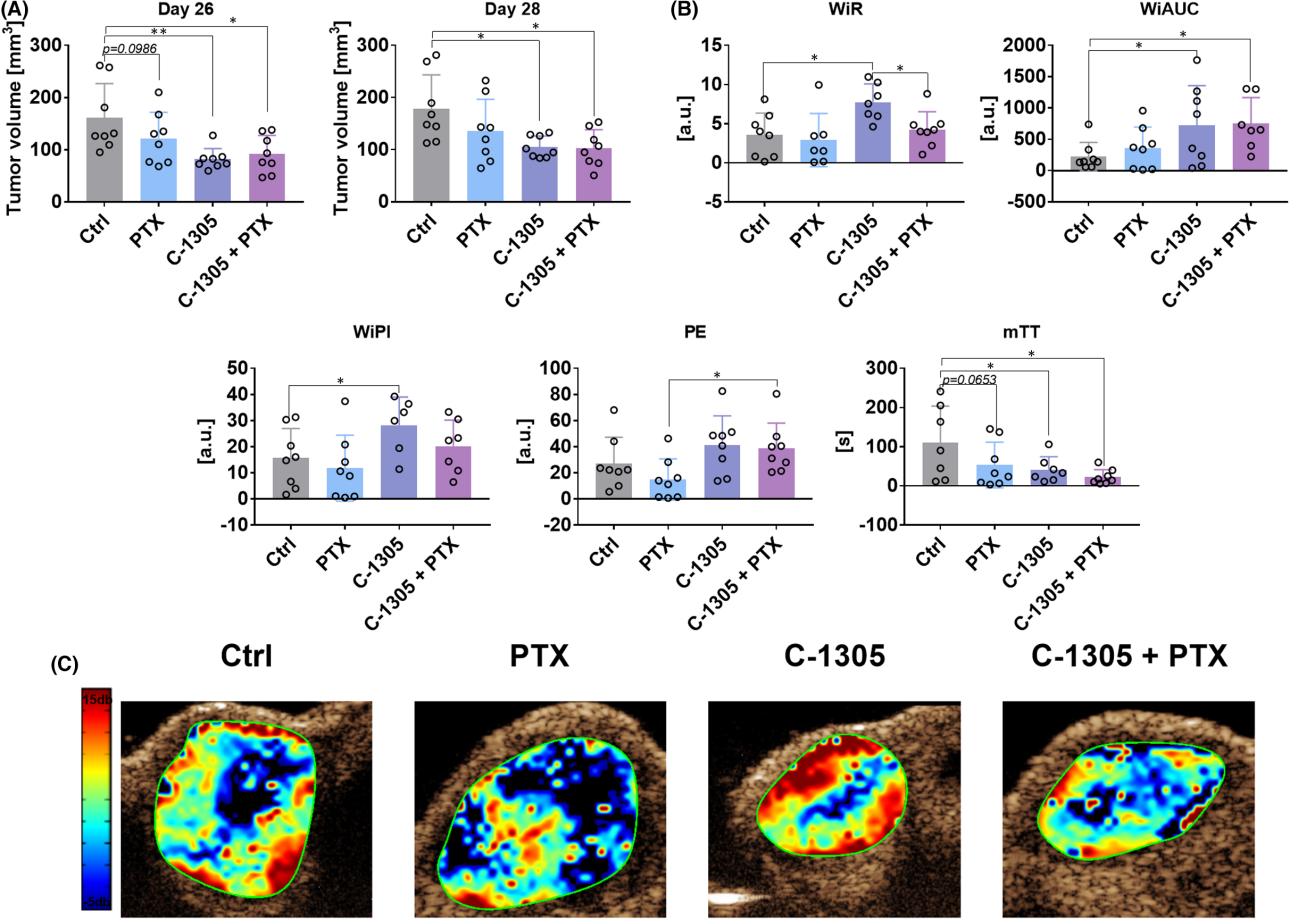
Tumour burden and blood flow in mice bearing human A549 lung cancer cells and treated with C‐1305 and PTX. (A) Tumour volume measured on Days 26 and 28. Tumour growth kinetics is presented in Figure S2. (B) Parameters describing blood flow in tumour tissue. WiR, wash‐in‐rate; WiAUC, wash‐in area under the curve; WiPI, wash‐in perfusion index; PE, peak enhancement; mTT, mean transit time. (C) Representative images of WiR. A549 cells were injected subcutaneously. Treatment was started on the 10th day with intraperitoneal injections of 10 mg/kg PTX and 30 mg/kg C‐1305. Then, PTX was injected once every week and C‐1305 was injected 5 days a week for a period of 3 weeks. For blood flow analysis on the 32nd day, anaesthetised mice were injected with a contrast agent and ultrasound detection was performed. *N* = 8; statistical analysis: Sidak's multiple comparisons test. *p < 0.05; **p < 0.01

Paclitaxel and C‐1305 when used alone did not significantly affect the growth of human colon cancer HCT116 cells. When combined treatment was given, only temporal (on the 17th day) inhibition of tumour growth was noticed (Figure 1B). We also included in our studies the time‐intensity curves (TIC) parameters describing the condition of blood flow in tumour tissue: peak enhancement (PE) representing the maximum intensity in the TIC curve (blood volume), time to peak (TTP)—time from zero intensity to maximum intensity, mean transit time (mTT) corresponding to the centre of gravity of best‐fit function of echo‐power (or fitted signal), wash‐in area under the TIC curve (WiAUC), wash‐in rate, maximum slope (WiR) corresponding to the pace of blood inflow, wash‐in perfusion index (WiPI = WiAUC/rise time (RT, calculated from the beginning of enhancement to PE))—representing the overall blood flow. Interestingly, the combined treatment led to a significant increase in wash‐in‐rate (WiR), wash‐in perfusion index (WiPI) and peak enhancement parameters, indicating an enhanced blood inflow into the tumour tissue. On the contrary, mean transit time (mTT) was decreased in all the treatment groups, suggesting a very slow reperfusion process (blood inflow from the adjacent tissue) (Figure 1C,D). In A549 human lung cancer model, tumour growth was significantly inhibited when C‐1305 was given alone and in combination with PTX (Figure 2A). In this tumour model, the impact of C‐1305 on tumour blood perfusion was also evident. C‐1305 significantly increased WiR, WiPI and wash‐in area under the curve (WiAUC). WiAUC was also found to be increased in the combined treatment group. On the contrary, a significant decrease in WiR was observed in the combined treatment group when compared to the group injected with C‐1305 alone. The inconsistent results between models can be explained by the differences in tumour growth kinetics and the size of tumours during perfusion measurements, resulting in, for example, different tumour tissue densities. Similar to HCT116 tumour model, mTT was found to be decreased in all treatment groups (Figure 2B,C).

### 3.2 Immunohistochemical analysis of tumour tissue

**FIGURE 3:**
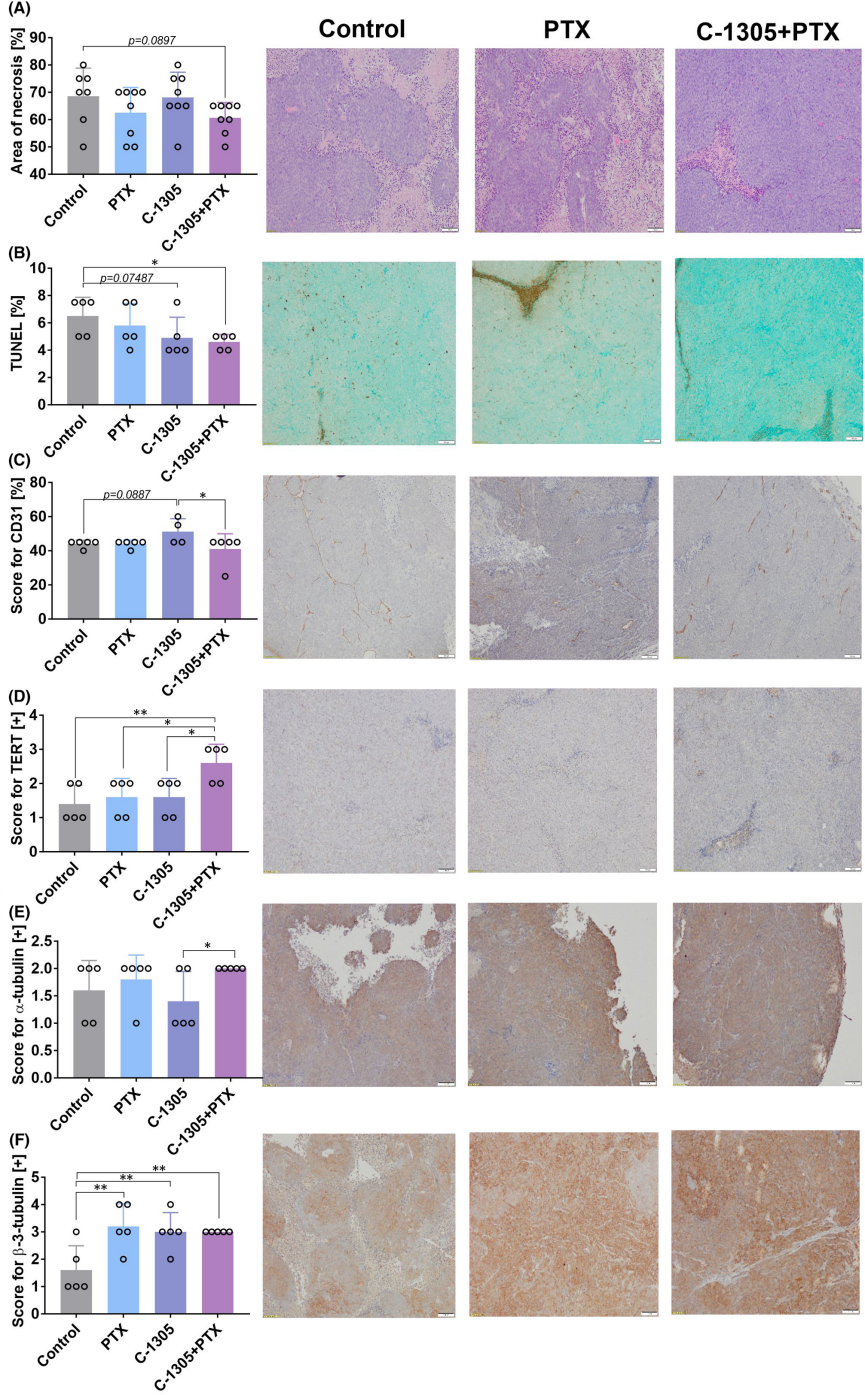
Histopathological analysis of tumour tissue from HCT116 tumour‐bearing mice. (A) Area of necrosis. (B) TUNEL staining (intensity of protein expression in nuclei and apoptotic bodies). (C) CD31 expression (ratio of the number of positively expressed vessels to the group of neoplastic cells as a percentage). Expression of (D) TERT, (E) α‐tubulin and (F) β‐3‐tubulin.( D–F) Scoring of staining intensity: + low, ++ medium, +++ high, ++++ high plus. Representative images of tumours procured from control mice treated with PTX and C‐1305 combined with PTX. Scale bar = 100 μm. Tumour tissue was harvested on Day 26. HCT116 cells were injected subcutaneously. Treatment was started on the 10th day with intraperitoneal injections of 10 mg/kg PTX and 30 mg/kg C‐1305. Then, PTX was injected once every week and C‐1305 was injected 5 days a week for a period of 3 weeks. *N* = 5; statistical analysis: Sidak's multiple comparisons test. *p < 0.05; **p < 0.01

**FIGURE 4:**
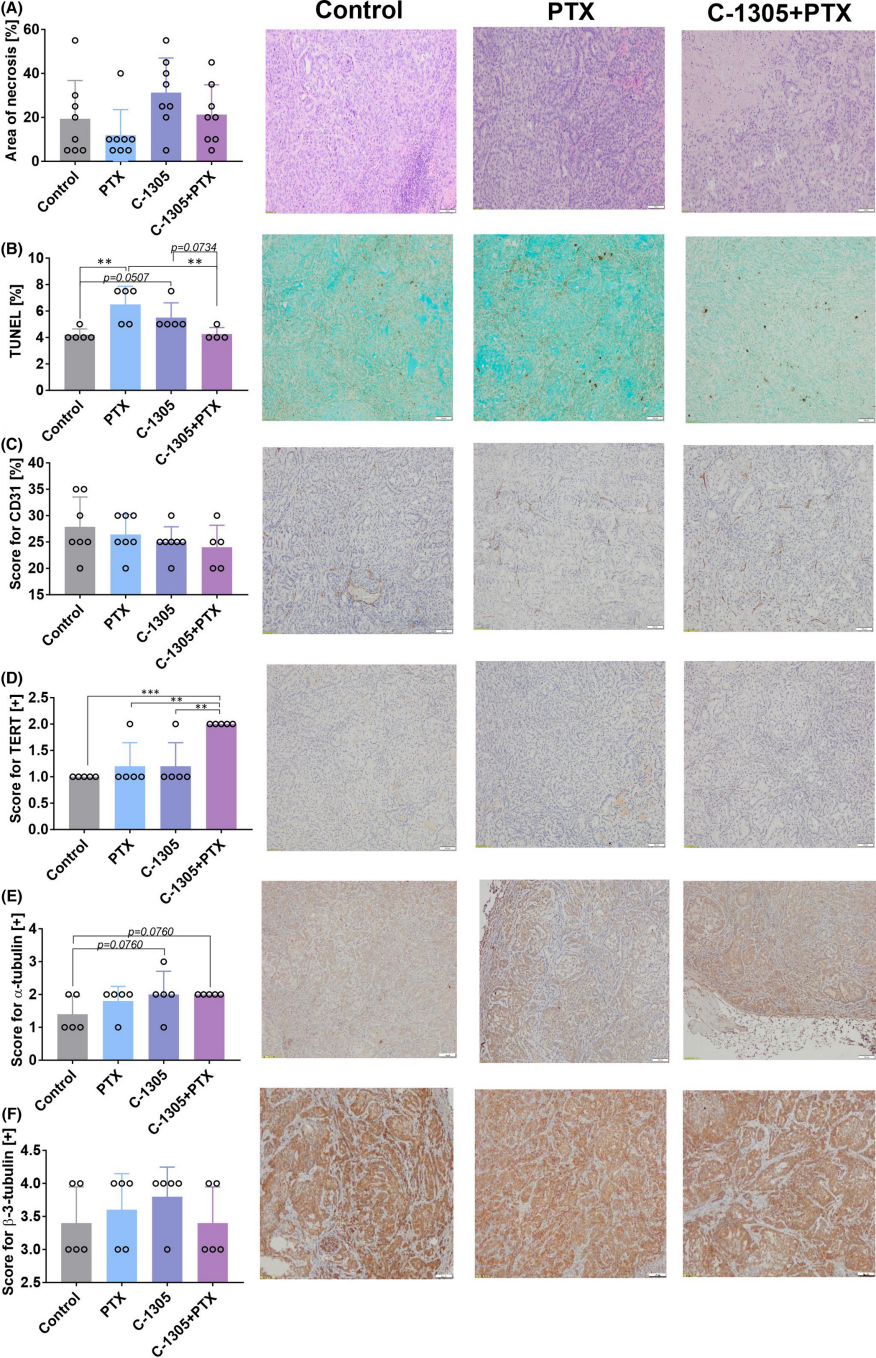
Histopathological analysis of tumour tissue from A549 tumour‐bearing mice. (A) Area of necrosis. (B) TUNEL staining (intensity of protein expression in nuclei and apoptotic bodies). (C) CD31 expression (ratio of the number of positively expressed vessels to the group of neoplastic cells as a percentage). Expression of (D) TERT, (E) α‐tubulin and (F) β‐3‐tubulin. (D–F) Scoring of staining intensity: + low, ++ medium, +++ high, ++++ high plus. Representative images of tumours obtained from control mice treated with PTX and C‐1305 combined with PTX. Scale bar = 100 μm. Tumour tissue was harvested on Day 34. HCT116 cells were injected subcutaneously. Treatment was started on the 10th day with intraperitoneal injections of 10 mg/kg PTX and 30 mg/kg C‐1305. Then, PTX was injected once every week and C‐1305 was injected 5 days a week for a period of 3 weeks. *N* = 5; statistical analysis: Sidak's multiple comparisons test. *p < 0.05; **p < 0.01

The tumours harvested from mice were analysed by histological and immunohistochemical staining techniques. Necrotic areas were limited in the mice bearing HCT116 tumours and treated with a combination of C‐1305 and PTX (Figure 3A), but a similar finding was not observed in A549 tumours (Figure 4A). TUNEL staining was the lowest in combined treatment group of mice bearing HCT116 tumours (Figure 3B), while in mice bearing A549 tumours, TUNEL staining was significantly lower in C‐1305 + PTX group when compared to PTX‐alone group. The administration of PTX alone increased TUNEL staining significantly in A549 tumour tissues (Figure 4B). CD31 staining was decreased in HCT116 tumours obtained from mice treated with combined treatment when compared to those collected from mice treated with C‐1305 alone (Figure 3C). In A549 tumours, the applied treatment did not significantly affect this parameter (Figure 4C). TERT expression increased significantly in HCT116 (Figure 3D) and A549 (Figure 4D) tumours extracted from mice treated with C‐1305 + PTX when compared to all other treatment groups. The staining for α‐tubulin was higher in HCT116 tumours obtained from the combined treatment group when compared to those procured from C‐1305‐alone group (Figure 3E). In mice bearing A549 tumours, the tendency toward increased expression of α‐tubulin was also observed in groups treated with a combination of C‐1305 and PTX (Figure 4E). The expression of β‐3‐tubulin in HCT116 tumours was significantly increased in all the treatment groups when compared to that observed in the control group (Figure 3F). No significant changes in β‐3‐tubulin expression were noticed in A549 tumours subjected to all treatments (Figure 4F).

### 3.3 Impact of treatments used on selected molecules and signalling pathways related to angiogenic process

**FIGURE 5:**
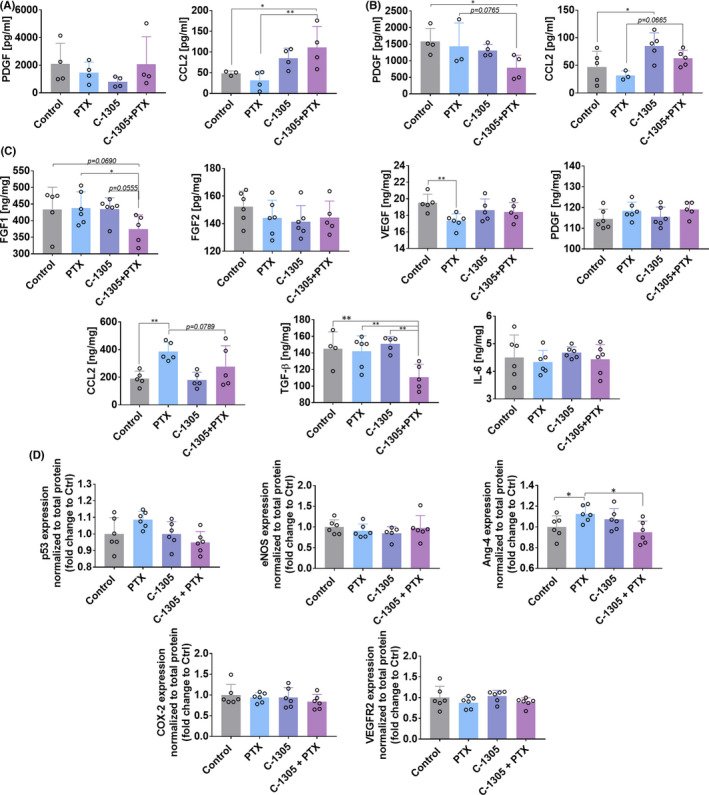
Expression of selected proteins in plasma and tumour tissue of treated mice. (A) Plasma of A549 tumour‐bearing mice. (B) Plasma of HCT116 tumour‐bearing mice. (C) Analysis of HCT116 tumour tissue by ELISA. (D) Analysis of HCT116 tumour tissue by Western blot. PDGF (AA) and CCL2 proteins were determined in plasma obtained from mice bearing (A) A549 and (B) HCT116 tumours and in lysates obtained from HCT116 tumours (C). The levels of VEGF, PDGF, CCL2, FGF1, FGF2 and IL‐6 were detected using ELISA according to the manufacturer's protocol. (D) The levels of Ang‐4, COX‐2, p53, VEGFR2 and eNOS were determined according to the procedure mentioned in the Jess user guide from ProteinSimple. After the detection of targeted proteins by chemiluminescence or NIR fluorescence, the primary and secondary antibodies were removed from capillaries to evaluate the total protein content in a single run. Compass software was used to program in the Jess language and analysis of the results. Quantification was performed by densitometry by calculating the area of targeted proteins and normalizing to the total protein content. Results are expressed as fold change in the expression of proteins when compared to control. *N* = 6. Statistical analysis: Sidak's multiple comparisons test. *p < 0.05; **p < 0.01

Earlier transcriptomic studies by Króliczewski et al.^1^ have shown that C‐1305 can modulate the expression of genes related to the angiogenesis process. Therefore, for further studies of plasma and tumour tissues, we chose known molecules that influence this process and the signalling pathways they activate. The plasma levels of IL‐6, VEGF, PDGF and CCL2 were assessed using ELISA. IL‐6 and VEGF were undetectable in the plasma of both HCT116 and A549 tumour‐bearing mice. The plasma level of PDGF did not change significantly in HCT116 tumour‐bearing mice (Figure 5A). In A549 tumour model, the PDGF level was significantly decreased in the combined treatment group (Figure 5B). The plasma level of CCL2 was found to be increased in mice bearing HCT116 tumours and treated with PTX + C‐1305 (Figure 5A). Similar results were observed in A549 tumours, but in this model, C‐1305 enhanced the plasma level of CCL2 significantly (Figure 5B).

The tumour tissue harvested from HCT116 tumour‐bearing mice was further analysed by ELISA (Figure 5C) and Western blot (Figure 5D). The lowest level of FGF1 was noticed in HCT116 tumours procured from the combined treatment group, whereas the FGF2 level did not show any significant change. The level of VEGF in tumour tissue was decreased followed by PTX treatment, whereas the PDGF level did not change significantly in the treated mice. PTX administration also resulted in an increase in the level of CCL2. The level of TGF‐β in the tumour tissue was significantly decreased in the combined treatment group when compared to all other treatment groups of mice. IL‐6 levels did not exhibit any changes in HCT116 tumours following the treatments (Figure 5C).

**FIGURE 6:**
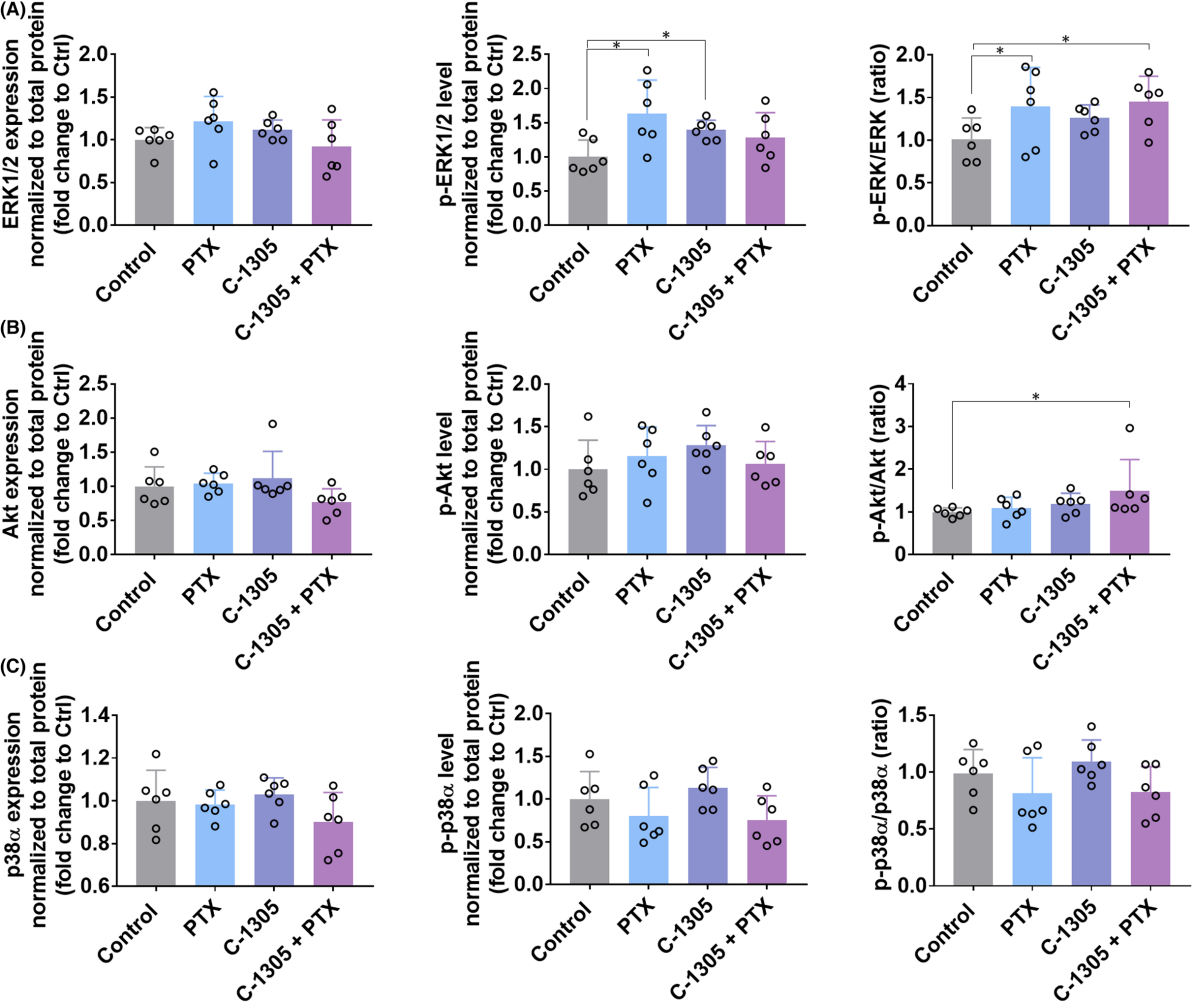
The analysis of ERK1/2, Akt and p38α phosphorylation and expression in HCT116 tumour lysates. Expression of phosphorylated forms of (A) ERK1/2, (B) Akt and (C) p38α. Jess Simple Western System, an automated capillary‐based size‐sorting system, was used according to the Jess user guide from ProteinSimple. After targeted protein detection by chemiluminescence or NIR fluorescence, the primary and secondary antibodies were removed from capillaries to evaluate the total protein content in a single run. Compass software was used to program in the Jess language and analysis of the results. Quantification was performed by densitometry by calculating the area of targeted proteins and normalizing to the total protein. Results are expressed as fold change in the expression of proteins when compared to control. *N* = 6. Statistical analysis: Dunnett's multiple comparisons test. *p < 0.05

Western blot analysis of HCT116 tumour tissue was performed to estimate the expression levels of eNOS, Ang‐4, COX‐2, VEGFR2, p53 (Figure 5D), ERK1/2, Akt and p38α (Figure 6). The results indicated that the treatments used did not affect the expression of p53, eNOS, COX‐2 and VEGFR (Figure 5D). The expression of Ang‐4 was diminished in the combined treatment group when compared to the PTX‐alone group. In addition, administration of PTX alone increased the expression of this protein (Figure 5D). The ratio of p‐ERK1/2 to total ERK1/2 was increased significantly by PTX, and a similar expression was observed in the combined treatment group (Figure 6A). p‐Akt/Akt ratio was increased significantly in the C‐1305 + PTX group (Figure 6B). Treatment of mice with both the compounds did not affect the expression of p38α (Figure 6C).

### 3.4 Effect of C‐1305 on human endothelial cells in vitro

**FIGURE 7:**
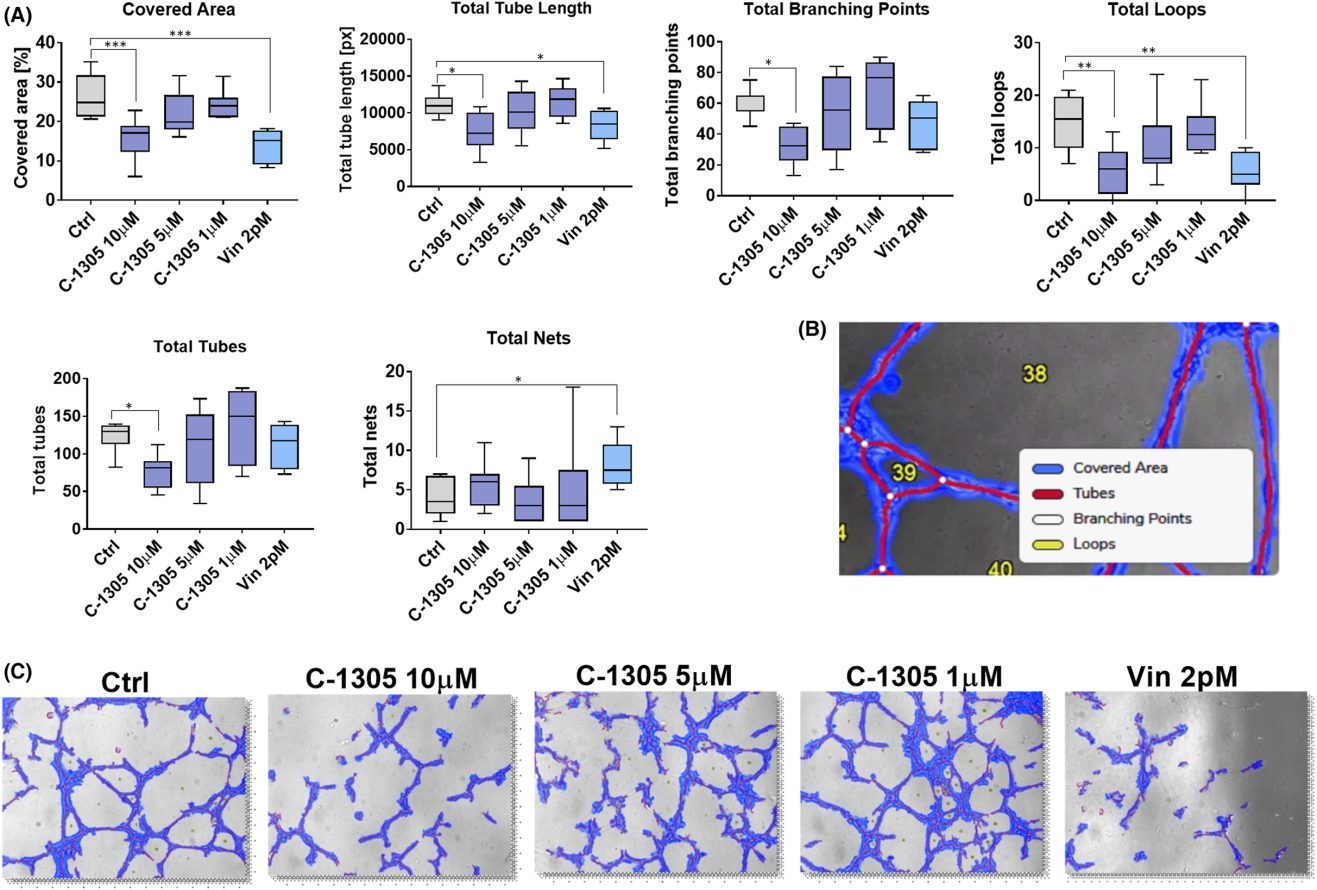
C‐1305 decreased the formation of capillary‐like structures in vitro. (A) The statistical analysis of parameters measured. (B) The legend for measurements performed. (C) The representative images. *N* = 8 for control and C‐1305 groups, and *N* = 6 for group injected with 2 pM vinblastine. Capillary‐like structure formations were accessed with the In Vitro Angiogenesis Assay Kit according to the manufacturer's protocol. Briefly, cells were passaged on matrigel‐coated 96‐well plates and treated with C‐1305 at final concentrations of 1, 5 and 10 μM, respectively. The number of capillary‐like structures was counted in each well (from six random focal fields at 20× magnification) after 8 h of incubation. A semiquantitative measurement of capillary structure formation was performed with the Wim Tube webserver. Statistical analysis: Dunnett's or Sidak's multiple comparison tests. *p < 0.05; **p < 0.01; ***p < 0.001

The effect of C‐1305 on capillary‐like structure formations in vitro was accessed. The results showed that when the highest concentration (10 μM) of C‐1305 was used, a significant decrease in the values of almost all the measured parameters, that is, covered area, total tube length, total branching points, total loops and total tubes formation, was observed (Figure 7).

### 3.5 Toxicological outcomes of treatments used

The body weight of mice treated with PTX alone and C‐1305 + PTX decreased during the course of the study, but the decrease did not exceed 10% (Figures S1 and S2). A change was observed in the biochemical parameters of blood only in mice bearing A549 tumours, whereas in combined treatment groups, a decrease in the levels of ASTL, CRE2, UREL and ALB was noticed (Figure S3).

## 4 DISCUSSION

C‐1305 is an imidazoacridinone analogue that has been described as an agent with potential antitumor activity and is known to inhibit the growth of murine B16 melanoma cells and C26 and C38 colon adenocarcinoma cells.^4^ Notably, only C‐1311 analogue of these compound passed Phase I clinical trials in patients with sarcomas and epithelial malignancies,^17^, ^18^ and Phase II clinical trials showed that this compound effectively controlled the disease in 40% of breast cancer patients who were refractory to taxanes, anthracyclines and various hormone and cytotoxic therapies.^15^ Furthermore, recent studies revealed that C‐1311 facilitated the restoration of the DNA damage response cascade in androgen‐dependent prostate cancer cells. In contrast, C‐1311 targeted cellular metabolism and inhibited the genes responsible for the regulation of glycolysis and gluconeogenesis pathways in androgen‐independent prostate cancer cells.^21^ Moreover, both compounds, C‐1305 and C‐1311 are selective inhibitors of cytochrome P450 (CYP) 1A2 and 3A4 isoenzymes.^22^, ^23^ In contrast, another study showed that C‐1305 strongly induced the activity and expression of CYP3A4 and CYP2C9, as well as expression of UDP‐glucuronosyltransferase (UGT) 1A1 and MDR1 in pregnane X receptor‐dependent HepG2 cells.^24^, ^25^ Overexpression of CYP2A4 enhanced the apoptosis induced by C‐1305 or C‐1311 in CHO cells.^26^, ^27^ Some authors suggest that extrahepatic UGT1A10 also plays an important role in the metabolism and bioactivation of C‐1305, and this biotransformation leads to enhanced proapoptotic activity of these glucuronide products.^28^, ^29^ Interestingly, although both C‐1305 and C‐1311 undergo metabolic transformation to the glucuronidated forms upon overexpression of UGT1A10, UGT1A10 overexpression significantly increased the cytotoxicity of C‐1305 but not C‐1311.^28^, ^29^ These findings contribute to the understanding of the mechanisms of action of both compounds and encourage further studies on the C‐1305 mode of action.

In addition, some studies focused on the safety profile of C‐1305, including therapeutic index and drug metabolism. Previous in vivo studies described that the maximum tolerated dose for C‐1305 in mice was 150 mg/kg when administered as a single injection or 250 mg/kg when the dosage was split and given as daily intraperitoneal injections for 5 days a week. The latter administration regimen produced less severe side effects in mice and showed a better therapeutic index when compared to the single administration of a higher dose of the drug.^4^ Studies that focused on the metabolism of C‐1305 indicated that the flavin‐containing monooxygenases, FMO‐1 and FMO‐3, are responsible for its biotransformation in liver microsomes and human hepatoma cells,^30^ the same as in the case of C‐1311.^23^ The studies on the metabolite profiles of C‐1305 revealed that C‐1305 underwent electrochemical oxidation primarily on the dialkylaminoalkylamino moiety. In addition, an unknown *N*‐dealkylated and hydroxylated derivatives of C‐1305 have been identified.^31^

However, there have been contradictory reports in the literature regarding the mechanism of apoptosis induced by C‐1305. Our recent study used next generation sequencing to identify signalling pathways that were induced in A549 and HCT116 cancer cells under the influence of C‐1305 showed that C‐1305 promotes direct microtubule stabilization as part of the mechanism of its action, thus leading to cell apoptosis.^1^ Furthermore, C‐1305‐promoted cell cycle arrest in the G_2_ phase is well reflected by the gene expression patterns.^1^ It was shown, inter alia, that the use of 1 μM PTX together with 3 μM C‐1305 produces the same effect as that observed with the 5 μM PTX alone, which was confirmed by analysing their effect on the tubulin polymerization rate. The results revealed that both compounds accelerated tubulin polymerization. Thus, the findings of these studies suggested a new mechanism of action for C‐1305, that is, disruption of normal cell division when combined with PTX, which provided a strong basis for conducting further research studies on the effects of C‐1305 compound.^1^

Our in vivo studies, that has been performed using the same cell lines as in in vitro approach, have shown that treatment with C‐1305 alone exhibits antitumor effect in A549 tumour model only, while no improvement in the effect was observed in the combined treatment group. In addition, no significant effect of the treatment on the expression of α‐ or β‐3‐tubulin was noticed in this tumour. However, combined treatment with both drugs showed a tendency to decrease the tumour growth in the HCT116 human colon cancer bearing mice. In this model, the α‐tubulin score was elevated in the combined treatment group when compared to the score observed in the group treated with C‐1305 alone. An increase in the staining intensity of β‐3‐tubulin was noticed in all the treated mice. The results of clinical trials indicate that β‐3‐tubulin could function as a negative prognostic factor but not as a predictive factor in the treatments involving the administration of PTX.^32^ In addition, β‐3‐tubulin serves as a downstream target of HDAC3 and mediates resistance to microtubule‐targeting drugs.^33^ Although the results with respect to the tumour growth were not satisfactory, we observed improved blood perfusion in both A549 and HCT116 tumours following treatment with C‐1305 alone and in the HCT116 tumours following combined treatment with both drugs. Increased staining of CD31 endothelium marker was also observed in HCT116 tumours in C‐1305‐alone group when compared to combined treatment group. Tumour angiogenesis is an important target for various anticancer therapies, and improved blood perfusion with well‐organized blood vessel network is important for the efficient delivery of the drug within the tumour tissue.^34^ The in‐depth analysis of the results of previous genome‐wide mRNA studies also suggests the impact of C‐1305 on the signalling pathways involved in angiogenesis.^1^ Among all the molecules analysed in the plasma of mice, a decreased level of PDGF was noticed in C‐1305 + PTX group (mice bearing HCT116 tumours). In the tumour tissue of the same mice, decreased levels of FGF1, TGF‐β and Ang‐4 were also observed. The combined treatment was associated with increased phosphorylation of ERK1/2 and Akt. Also, an increase in the plasma level of CCL2 was noticed, and a similar tendency was observed in HCT116 tumour tissue. CCL2 chemokine was found to be directly involved in the process of tumour angiogenesis, and its effects are dependent on the ERK1/2 signalling cascade.^35^ This finding may explain the reason behind the increased ERK1/2 phosphorylation levels observed in our studies. However, decreased levels of other proangiogenic molecules, such as FGF1,^36^ TGF‐β^37^ and Ang‐4,^38^ may indicate a modulating effect of the combined treatment of C‐1305 and PTX on the angiogenesis process. It should also be emphasized that C‐1305 inhibited angiogenesis in an in vitro assay.

## 5 CONCLUSION

C‐1305 and C‐1311 are considered to be the representatives of the imidazoacridinone analogues, and C‐1311 (Symadex™, NSC‐645809) compound entered the clinical trials.^15^, ^17^, ^18^ Although previous in vitro and in vivo studies showed encouraging results in human colon and lung cancers when combined treatment with PTX was given, the in vivo experiments of the present study showed that the antitumor activity was restricted and associated with the modulation of tumour angiogenesis.

## AUTHOR CONTRIBUTIONS

**Marta Świtalska:** Data curation (lead); formal analysis (lead); investigation (lead); methodology (lead); writing – original draft (supporting). **Beata Filip‐Psurska:** Data curation (lead); formal analysis (lead); investigation (lead); methodology (lead); writing – original draft (supporting). **Magdalena Milczarek:** Data curation (lead); formal analysis (lead); investigation (lead); methodology (lead); writing – original draft (supporting). **Mateusz Psurski:** Data curation (supporting); investigation (supporting); methodology (supporting); software (supporting); writing – original draft (supporting). **Adrianna Moszyńska:** Data curation (supporting); investigation (supporting); methodology (supporting); software (supporting); writing – original draft (supporting). **Aleksandra M. Dąbrowska:** Investigation (supporting); writing – original draft (supporting). **Małgorzata Gawrońska:** Investigation (supporting); writing – original draft (supporting). **Karol Krzymiński:** Investigation (supporting); writing – original draft (supporting). **Maciej Bagiński:** Conceptualization (equal); funding acquisition (equal); project administration (equal); resources (equal); supervision (equal); writing – review and editing (equal). **Rafał Bartoszewski:** Conceptualization (equal); funding acquisition (equal); project administration (equal); resources (equal); supervision (equal); writing – review and editing (equal). **Joanna Wietrzyk:** Conceptualization (lead); data curation (supporting); project administration (equal); resources (equal); supervision (equal); writing – original draft (lead); writing – review and editing (lead).

## CONFLICT OF INTEREST

The authors confirm that there are no conflicts of interest.

## Supporting information


Figure S1‐S3

## Data Availability

The data supporting the findings of this study are available in the supplementary material of this article.
