# Supplementary material for: Combined anticancer therapy with imidazoacridinone analogue C‐1305 and paclitaxel in human lung and colon cancer xenografts—Modulation of tumour angiogenesis
Source: J Cell Mol Med. 2022 Jun 14;26(14):3950–64. doi: 10.1111/jcmm.17430 (PMC9279600; doi:10.1111/jcmm.17430)
Supplement: Supplementary file 1 — Figure S1‐S3 [file JCMM-26-3950-s001.docx]

Supplementary Material:

**Combined anticancer therapy with imidazoacridinone analog C-1305 and paclitaxel in human lung and colon cancer xenografts**—**modulation of tumor angiogenesis**

Marta Świtalska^1^, Beata Filip-Psurska^1^, Magdalena Milczarek^1^, Mateusz Psurski^1^, Adrianna Moszyńska^2,3^, Aleksandra Dąbrowska^4^, Małgorzata Gawrońska^4^, Karol Krzymiński^4^, Maciej Bagiński^5^, Rafał Bartoszewski^2^, Joanna Wietrzyk^1^

^1^ Department of Experimental Oncology, Hirszfeld Institute of Immunology and Experimental Therapy, Wrocław, Poland

^2^ Department of Biology and Pharmaceutical Botany, Medical University of Gdansk, Al. Gen. J. Hallera 107, Gdańsk, Poland

^3^ Department of Immunology, Faculty of Biochemistry, Biophysics and Biotechnology, Jagiellonian University, Kraków, Poland

^4^ Faculty of Chemistry, University of Gdańsk, W. Stwosza 63, Gdańsk, Poland

^5^ Department of Pharmaceutical Technology and Biochemistry, Faculty of Chemistry, Gdansk University of Technology, Gdańsk, Poland


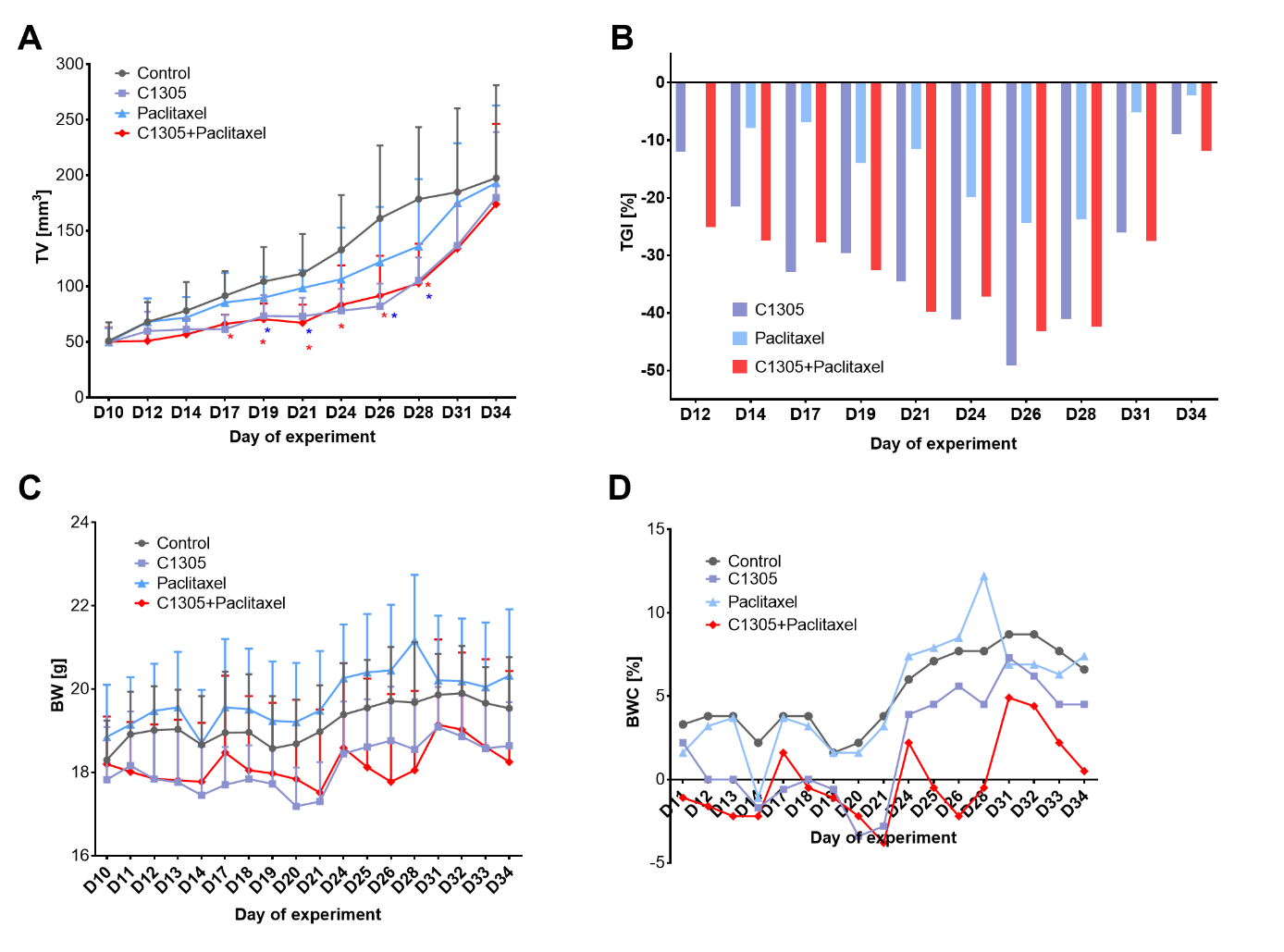


**Figure S1.** Tumor growth and body weight kinetics in mice bearing human HCT116 colon cancer cells and treated with C-1305 and PTX. **A**) Tumor volume measured during the experiment. **B**) Tumor growth inhibition (TGI) was calculated as $TGI \left[ \% \right]= 100-\left[ \left( \frac{{TV}_{X}^{D}}{{TV}_{ctrl}^{D}} \right)\times100 \right]$, where ${TV}_{X}^{D}$ refers to mean tumor volume in group X at day D and ${TV}_{ctrl}^{D}$ refers to mean tumor volume in control group at the same day. **C**) Body weight of HCT116 tumor-bearing mice. **D**) Body weight change (BWC) was calculated using the following formula: BWC (%) = [(body weight on the last day) − (body weight on day 0)]/(body weight on day 0) × 100 (%). HCT116 cells were injected subcutaneously. Treatment was started on the 10th day with intraperitoneal injections of 10 mg/kg PTX and 30 mg/kg C-1305. Then, PTX was injected every week and C-1305 was injected 5 days a week for a period of 3 weeks. N=8; statistical analysis: Sidak’s multiple comparisons test. **p*<0.05.


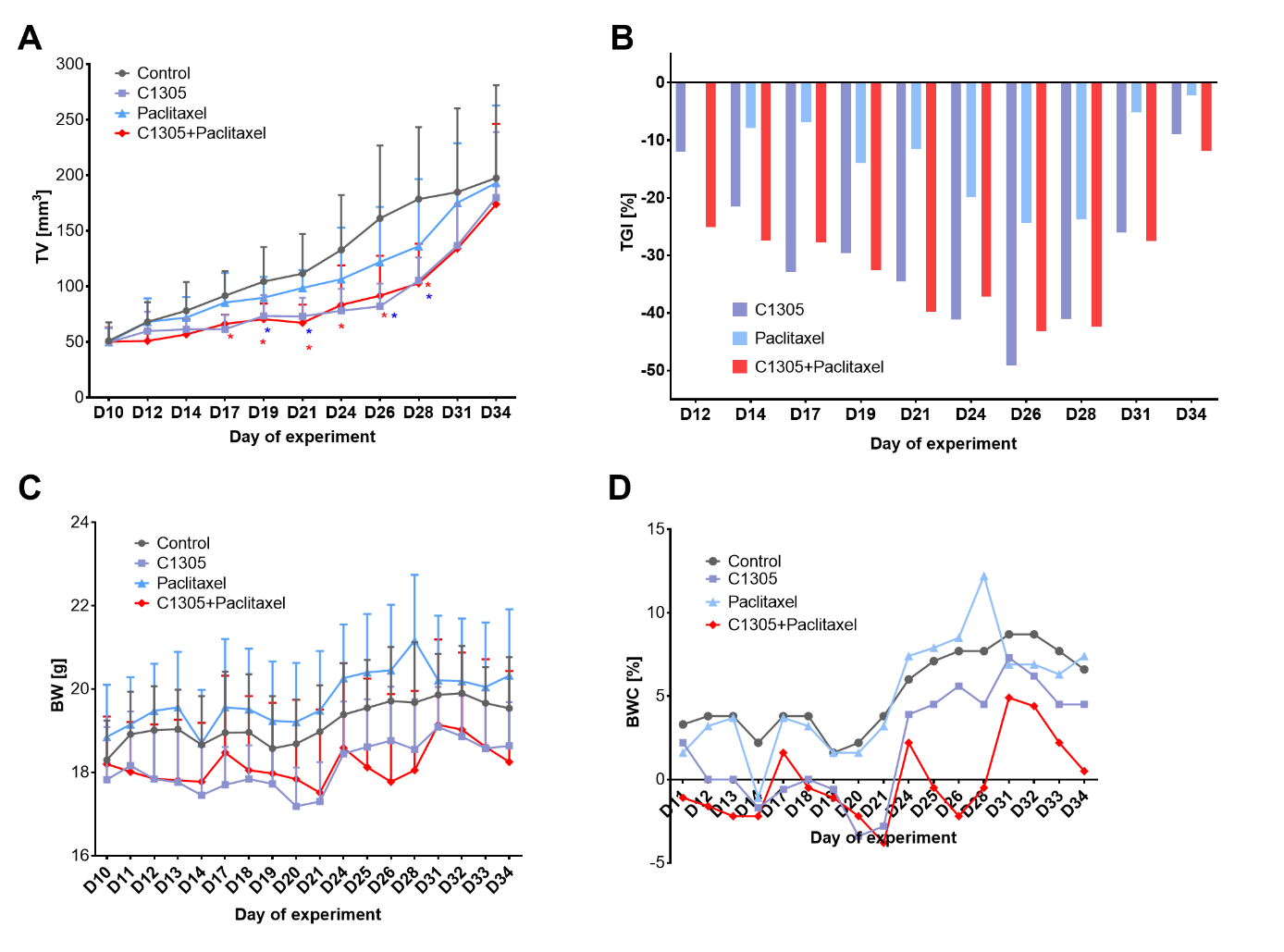


**Figure S2.** Tumor growth and body weight kinetics in mice bearing human A549 lung cancer cells and treated with C-1305 and PTX. **A**) Tumor volume measured during experiment. **B**) Tumor growth inhibition (TGI) was calculated as $TGI \left[ \% \right]= 100-\left[ \left( \frac{{TV}_{X}^{D}}{{TV}_{ctrl}^{D}} \right)\times100 \right]$, where ${TV}_{X}^{D}$ refers to mean tumor volume in group X at day D and ${TV}_{ctrl}^{D}$ refers to mean tumor volume in the control group on the same day. **C**) Body weight of HCT116 tumor-bearing mice. **D**) Body weight change (BWC) was calculated using the following formula: BWC (%) = [(body weight on the last day) − (body weight on day 0)]/(body weight on day 0) × 100 (%). A549 cells were injected subcutaneously. Treatment was started on the 10th day with intraperitoneal injections of 10 mg/kg PTX and 30 mg/kg C-1305. Then, PTX was injected every week and C-1305 was injected 5 days a week for a period of 3 weeks. N=8; statistical analysis: Sidak’s multiple comparisons test. **p*<0.05.


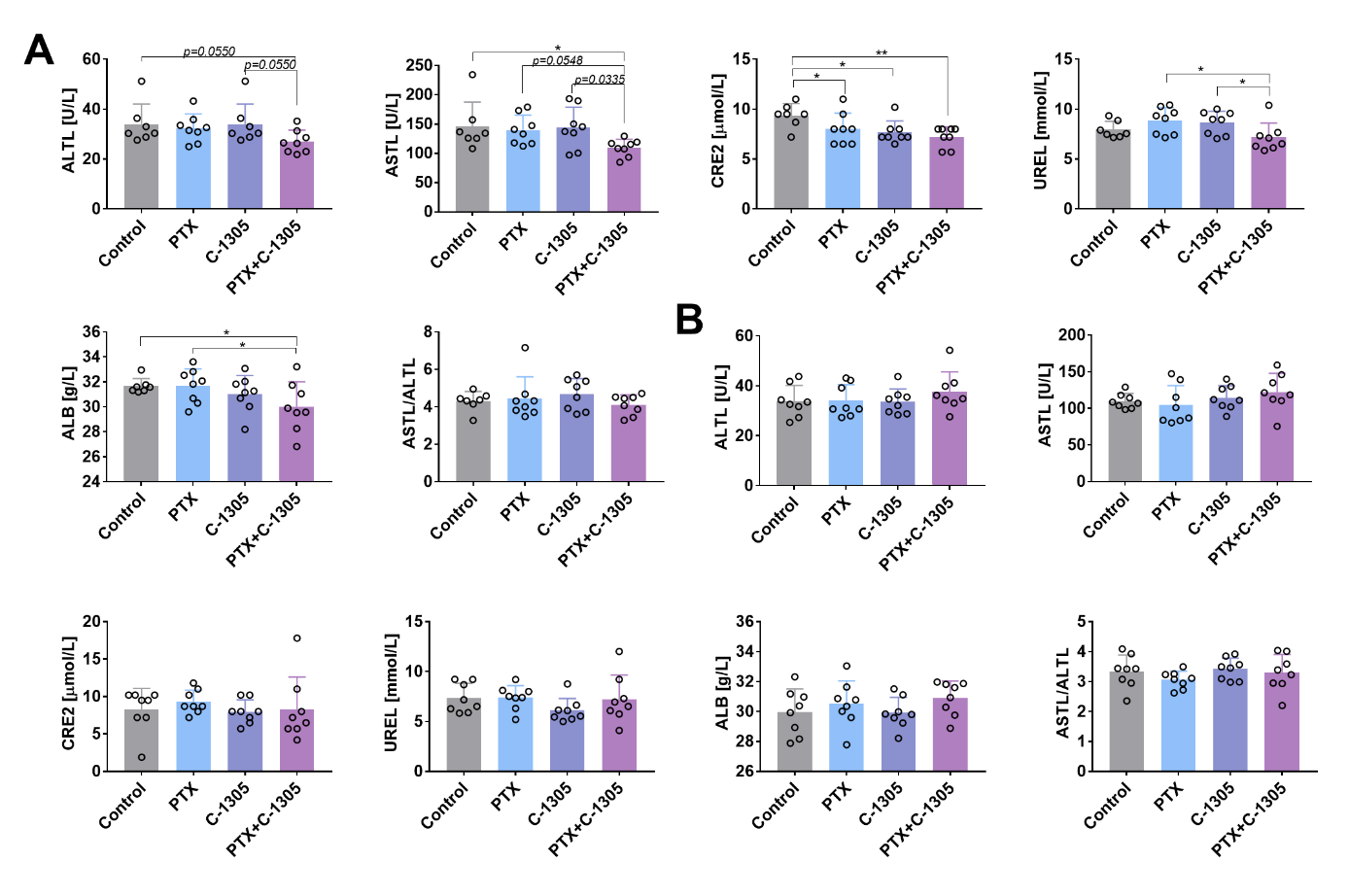
 **Figure S3.** Biochemical analysis of plasma obtained from mice bearing (**A**) A549 and (**B**) HCT116 tumors. The plasma levels of ALTL, ASTL, UREL, CRE2, and ALB were evaluated in Cobas C 111 analyzer using reagents and procedures provided by the manufacturer. The ASTL/ALTL ratio was calculated. N=8; statistical analysis: Sidak’s multiple comparisons test. **p*<0.05.

Fig. S3.
